# Practical aspects of electrophoretic deposition to produce commercially viable supercapacitor energy storage electrodes†

**DOI:** 10.1039/d0ra09197a

**Published:** 2021-06-09

**Authors:** Barun Kumar Chakrabarti, Chee Tong John Low

**Affiliations:** WMG, Warwick Electrochemical Engineering Group, Energy Innovation Centre, University of Warwick Coventry CV4 7AL UK Barun.Chakrabarti@warwick.ac.uk C.T.J.Low@warwick.ac.uk

## Abstract

Electrophoretic deposition (EPD) is a highly convenient and demonstrated industrial operation for the manufacture of surface coatings. Recent years are seeing increasing evidence in using this technique to produce energy storage electrodes (notably for lithium-ion batteries, solid-state devices, supercapacitors, and flow batteries), but their advancement for industrialisation remains unclear. Using activated carbon (AC) as an exemplary supercapacitor material, this study reports the practical aspects of porous energy storage electrodes produced by the EPD technique. Practical electrodes with commercially viable parameters are shown, specifically high density active material (in excess of 9.8 mg cm^−2^) and very thick coating layer (about 168 μm). Research investigations including colloidal electrolyte formulations, electrode deposition parameters and cell performance testing are reported. Materials and electrode properties were studied by various charactersisation tools. Prototype A7 sized pouch cells were assembled and tested to show evidence of practical EPD electrodes in a commercial cell format. Electrochemical performance of EPD over slurry casting is presented. In short, this research shows the successful production of practical EPD electrodes for electrochemical energy storage, which is directly relevant for scale-up industrial adoption and can be applied as a platform electrode manufacturing technology for any battery and supercapacitor materials.

## Abstract 2

Electrophoretic deposition (EPD) is a highly convenient and demonstrated industrial operation for coatings manufacture. It is now suitable for the production of practical energy storage electrodes for batteries, capacitors & solid-state devices.

## Graphical abstract

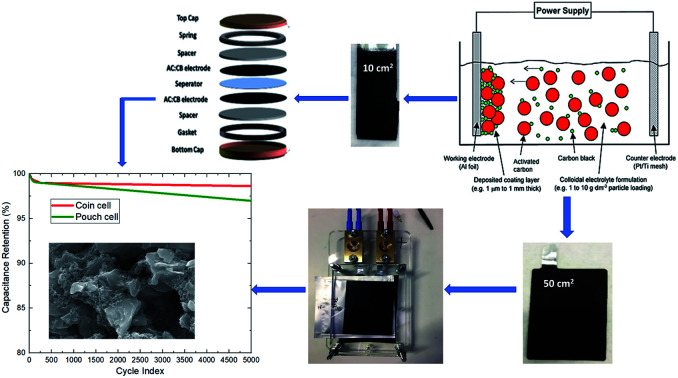

## Introduction

Electrophoretic deposition (EPD) has been employed in the electrophoretic paints industry since the 1970s. The high level of automation, low levels of pollution and homogeneity of deposited layers are advantages that led to the successful application of this technique for coating paints onto car bodies. It continues to offer a wealth of possibilities to deposit coatings with controllable features (thin or thick layers, 2D or 3D shapes, compact or porous, composite or graded or multi-layered). Although our understanding of the deposition mechanisms and fundamental manufacturing aspects is still far from complete, this has clearly not prevented the use of this highly versatile technology on an industrial scale.

In its simplest form, EPD exploits the direct interaction of charged particles in a colloidal electrolyte with an electric field. The charged particles migrate to a deposition substrate, then are deposited onto it and form a layer by deposits build-up. Recent years are seeing many published evidence in EPD for energy storage applications; notably lithium-ion battery electrode,^1^ solid-state electrolyte,^2^ membrane electrode assembly,^3^ supercapacitor^4^ and flow battery,^5^ but their advancement for industrialisation are far from actual adoption. An obvious reason is because the published research have only focused on depositing very thin layer (<1 μm), which gives the extreme performance values that can only be attributed to a complete utilization of low density active materials (1 mg cm^−2^) for fast accessibility of electrons and ions. While these fundamental studies are useful for identifying the maximum achievable properties, they are absolutely impractical for any commercial applications which demand thick layers (typically 50 to 200 μm) and high density active materials (typically 5 to 20 mg cm^−2^) to provide usable capacity for all power extraction capabilities. For more reading, several published articles on true performance metrics of supercapacitors are available.^6,7^

It is true for commercial supercapacitor (using activated carbon for capacitive energy storage) to account for the entire mass of the device, which include inactive materials such as current collector, separator, electrode, binder, electrolyte and packing. In a typical case, the mass of active material (*e.g.* 10 mg cm^−2^) is about 30% of the total weight of device. In such a case, device performance calculated from the electrode property would be reduced by a factor of 3 or 4, and this is raised to 30 if thinner electrode with lower mass loading (*e.g.* 1 mg cm^−2^) is used. High mass loading of active material is necessary for lowering the overheads contribution from inactive materials, but this often leads to reduced capacitance due to mass transport limitations. It is challenging to produce thick electrodes with high density active materials and good mechanical strength, especially delivering high energy density without sacrificing power density. Although supercapacitors are commercial devices, many discoveries and research innovation are continuing. The performance metrics and obstacles from transitioning lab to industrial operation are numerous.^8^

In view of closing the knowledge gap between fundamental studies and commercial applications, this study reports the practical aspects of porous energy storage electrodes produced by EPD technique. Practical electrodes with industrially relevant parameters are researched, specifically high mass loading of active material and thick coating layer. Activated carbon was used as an exemplary supercapacitor active material; it was chosen because all worldwide supercapacitor companies such as NESSCAP, Panasonic, Maxwell (now Tesla) and NEC use this material for the construction of commercial devices. Other allotropes of carbon such as graphene, carbon nanotube and composites are available in the development of future supercapacitors.^9,10^

Key investigations from this study include:

• Formulation of colloidal electrolyte recipes containing activated carbon particles,

• Methodologies and processes of EPD to make practical energy storage electrodes,

• Impact of electrode calendaring on cell performance,

• Electrochemical coin cell cycling activities, and

• Scaling-up EPD studies for pouch cell manufacture and their electrochemical testing activities.

Materials and electrodes were analysed by SEM (microstructure imaging), EDX (element mapping) and ion-milling (cross-section preparation). Electrochemical cycling performance of EPD electrodes over slurry casting are compared. The knowledge generated in this study are common across technological fields, and can be of direct relevance for systematic optimisation of any existing and future versions of lithium-ion batteries, solid-state components (electrode; electrolyte), supercapacitors and flow batteries. It is hopeful that this study would add new evidence in the growing versatility of EPD technology, especially designing and innovating this industrial manufacturing process for the modern electrochemical energy storage devices.

## Experimental

### Materials used in this study

All materials are commercially sourced. Activated carbon (YEC-8B, 8 to 10 μm, Fuzhou Yihuan Carbon, China) and carbon black (SuperP, 100 to 200 nm, 75 m^2^ g^−1^, Timcal) are used. Solvents studied are *N*-methyl-2-pyrrolidone (Acros), isopropyl alcohol (Merck) and acetone (Sigma-Aldrich). Binders are ethyl cellulose (Fisher) and polyvinylidene fluoride (Targray). Charge agents are magnesium chloride and iodine (Acros Organics).

### EPD electrode manufacture operation

Colloidal electrolyte has 3.3 g L^−1^ total materials loading, which contains 90 wt% active material (3.0 g L^−1^ activated carbon) and 10 wt% inactive material for electrical conductivity purpose (0.3 g L^−1^ carbon black). Polymer binder and charging agent are added to the electrolyte, then ultrasonicated for 2 hours prior to use. In the EPD reactor setup, Al foil as deposition substrate (15 μm thick, 7.5 cm by 2.5 cm) and Pt/Ti mesh as counter electrode (1 mm thick, 7.5 cm by 2.5 cm; 20 mg cm^−2^ Pt) are used. They are cleaned in IPA solution. Inter-electrode gap was 10 mm. Cathodic EPD is carried out at 70 V for 20 minutes. Electrolyte temperature is maintained at 40 °C and stirred at 300 rpm by a magnetic stirrer. Once EPD electrodes are formed, they are left to dry on a hotplate (40 °C, 10 min) and stored in a vacuum oven at 60 °C (overnight). In some cases, EPD electrodes are hot pressed (10 ton force, 85 °C to 70% of original thickness) using an electric hot rolling press (Gelon). Electrodes are also made by slurry casting (25% activated carbon, 3% carbon black, 1.5% PVDF, 70.5% NMP; this gives 29 wt% solid content).

### Coin cell manufacture and electrochemical testing

Coin cells (CR2032) are produced using processing tools and cell components from MTI Corporation. Celgard 2325 microporous membrane (25 μm thickness, PP/PE/PP layers) is the coin cell separator. 1 M tetraethylammonium tetrafluoroborate (99% purity, Sigma-Aldrich) dissolved in acetonitrile (99% purity, VWR) are used as supercapacitor electrolyte. Coin cells are kept at room temperature (overnight) to allow electrolyte soaking into porous structure of activated carbon. Electrochemical testing involves: cyclic voltammetry (0 V to 2.5 V; 20 mV s^−1^), galvanostatic tests (0.1 to 10 A g^−1^; 0 V and 2.5 V cut-off voltage for discharge and charge), and electrochemical impedance spectroscopy tests (0.01 Hz to 10 kHz; 10 mV amplitude). Using 10th cycle data, specific capacitance (*C*, F g^−1^), energy density (*E*, W h kg^−1^) and power density (*P*, W kg^−1^) are calculated.1
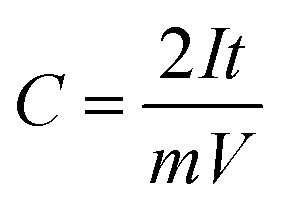
2
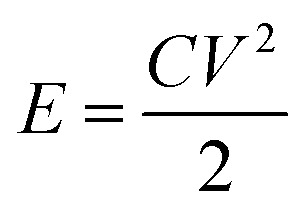
3
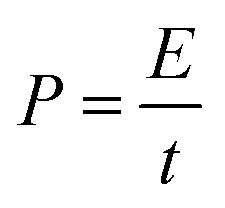
where *I*, *t*, *m* and *V* are applied current (A), discharge time (s), mass of deposits (g) and cell voltage window (V).

### Other characterization activities (material and electrode)

Surface charge of particles was measured by zeta potential (Malvern Zetasizer Nano ZS). Microstructures and elements mapping were produced by scanning electron microscopy equipped with EDX tools (Carl Zeiss Gemini). Cross-sectioning samples were produced by focused ion beam milling (Scios, FEI). Film thickness was measured by thickness gauge (Mitutoyo), and mass loading by SE2 ultra-microbalance (Sartorius). XPS (Omicron) was used for surface elemental composition. Specific surface areas were determined by BET analyser (IQ3 Quantachrome); pore volume was calculated from the amount of nitrogen adsorbed at 0.90 atm. and pore size distribution was calculated by QSDFT method using isothermal data as detailed in our recent investigation on redox flow battery.^5,11,12^

### Pouch cell manufacture and performance testing

Pouch cells (A7 size, single layer) are produced using industrial equipment (Sovema Group) including electrode cutting, tab welding, separator wrapping, electrolyte filling and packaging.

Electrolyte volume for pouch cell is estimated by:4
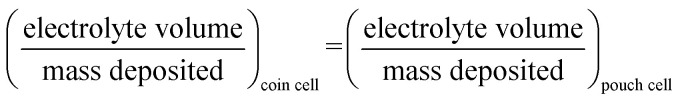


**Fig. 1:**
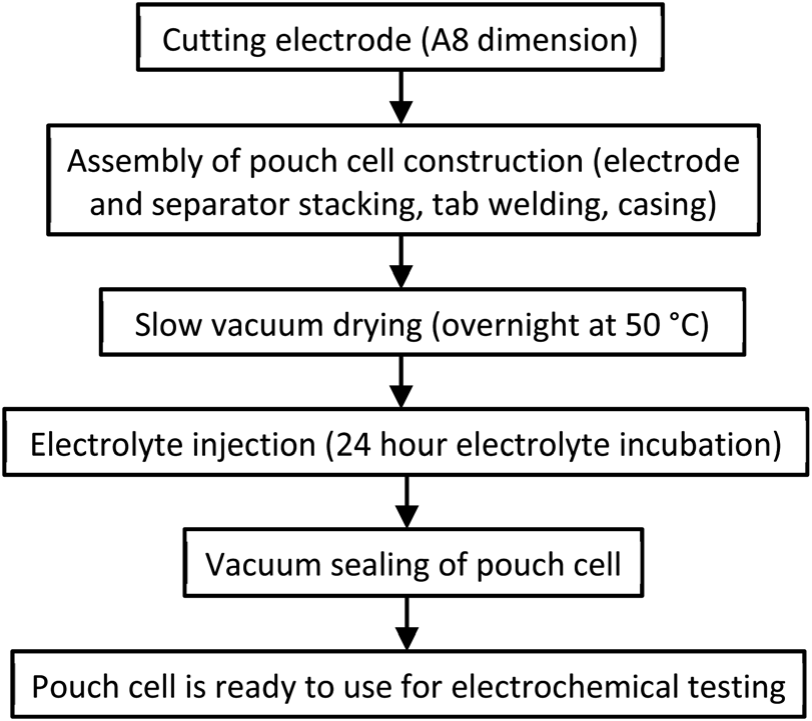
Pouch cell assembly manufacturing operation.

Prior to any testing, pouch cells are put under formation using Maccor Series 4000 (*i.e.* cycling between 0 and 2 V at 5 mV s^−1^). Electrochemical testing parameters for pouch cells are similar to those used in the coin cell activities, but performed at 7 kg compression test jig (to replicate similar compression condition as in the coin cells). Fig. 1 shows pouch cell assembly steps and their processing details.

## Results and discussion

Supercapacitor device consists of two energy storage electrodes that are isolated from electrical contact by a porous separator. Commercial devices have electrodes with sufficiently thick layers (50 to 200 μm) and high mass loadings (5 to 10 mg cm^−2^), in order to provide meaningful performance characteristics for practical applications. Very thin layer and low mass loading are impractical for real-world operations. All experiments in this study are therefore performed with EPD electrodes that can meet this important industrial criterion for practical energy storage.

### Equipment setup and manufacturing processes of EPD

**Fig. 2:**
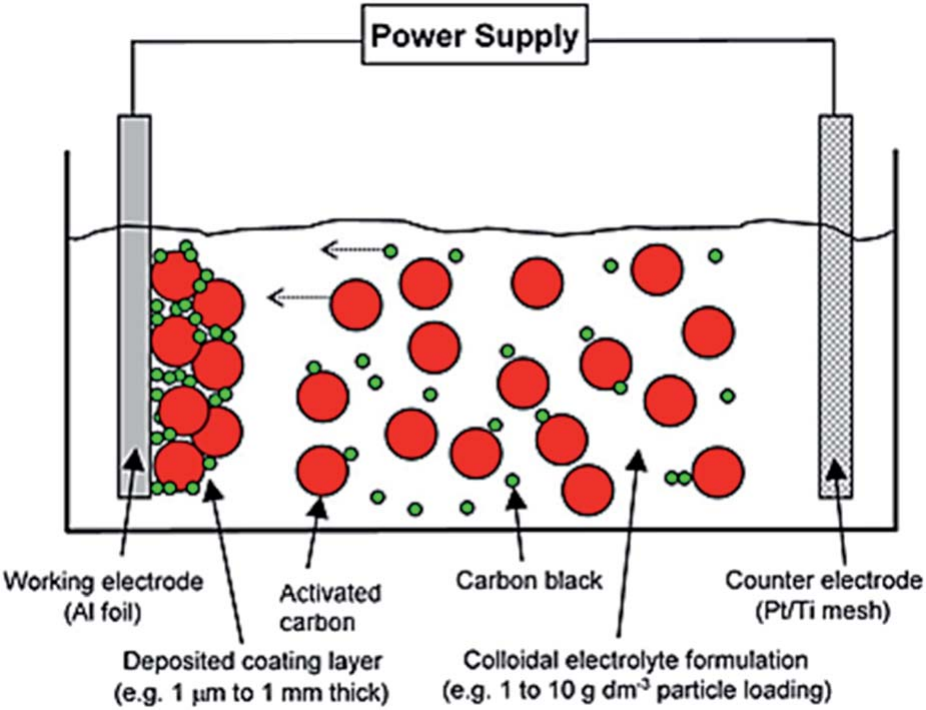
Representation on electrophoretic deposition (EPD) of activated carbon (8 to 10 μm) and carbon black (100 to 200 nm) onto Al foil (15 μm) to form a controllable coating film thickness (1 μm to 1 mm). Reproduced with permission from Wiley.^1^

A simple beaker setup was used (Fig. 2), which consists of two parallel facing electrodes. The essential components are:

#### • Working electrode

This is the deposition substrate where electrochemical materials, in this case activated carbon particles for supercapacitor, will be deposited. It is also the choice of current collector for the supercapacitor device, in this case Al foil.

#### • Counter electrode

This is an electrochemically inert current collector, in this case platinised/titanium (Pt/Ti) which is stable in the electrolyte, allowing electrons transfer rather than ions exchange.

#### • Power supply

This provides a source of high voltage, typically <100 V for electrophoretic deposition. Under the influence of an electrical profile between the two electrodes, the electrochemical materials migrate to the surface of the working electrode and are then deposited onto it forming a thick layer of deposits.

#### • Colloidal electrolyte

The solution contains activated carbon particles and the other associated chemicals and materials in its formulation, *e.g.* charging agent (to induce surface charge on electrochemical materials) and binder (to bind the materials together and provide adhesion onto the deposition substrate).

EPD electrode manufacture operation involves several steps:

(1) Prepare the colloidal electrolyte solution containing activated carbon particles and other materials.

(2) Pass an electrical current to deposit the particles onto the surface of deposition substrate (called working electrode), thereby forming a layer of deposits coating (called EPD electrode). Choice of deposition substrate: 2D foil, 3D mesh, 3D foam and 3D fibrous structures.

(3) Dry the EPD electrode (*i.e.* evaporating the liquid).

(4) Use EPD electrode directly, or calendar prior to use, in the assembly of coin, pouch and cylindrical cells.

### Colloidal electrolytes formulations

EPD electrode manufacture was commenced by choosing a suitable solvent (NMP, IPA, acetone) in the presence of charging agent (MgCl_2_, I_2_) and binder (PVDF, ethyl cellulose). NMP was an adaptation from slurry casting of lithium-ion battery materials. IPA and acetone are investigated because they are common laboratory solvents with distribution and disposal infrastructure, plus having sufficiently high dielectric constant and low viscosity for successful EPD operation. PVDF binder was used with NMP and acetone recipes, whilst ethyl cellulose binder was used in IPA. Charging agent MgCl_2_ was used in NMP and IPA, whilst I_2_ in acetone.

See Table 1 for a summary of the experiments.

**Table 1:** Impact of colloidal electrolyte formulations on 10 cm^2^ electrode manufacture and their properties. Choice of binders for NMP (0.05 g dm^−3^ PVDF), IPA (0.25 g dm^−3^ ethyl cellulose), acetone (0.01 g dm^−3^ PVDF). All EPD electrolytes are formulated with 3 g dm^−3^ total mass loading (80% activated carbon, 10% carbon black, 10% charging agent). Unless stated, the colloidal electrolyte was kept under 300 rpm and 40 °C; 70 V was applied for 20 minutes deposition and hot-pressed (calendared) EPD electrode (10 tonne force, 85 °C)

Choice of colloidal electrolyte parameters	EPD deposited thickness [μm]	Adhesion to Al foil (by physical observation)	Mass loading of deposited materials [mg cm^−2^]	Capacitance [F g^−1^] @ 0.1 A g^−1^
**Slurry cast (NMP)**	140	Good	8.27	107

**Types of solvent for EPD**				
NMP	109	Good	7.01	42.13
IPA	118	Marginal	5.92	58.00
Acetone	131	Very good	9.80	165.00

**Deposition duration (NMP)**				
20 min	109	Good	7.01	42.13
40 min	113	Good	7.69	35.54

**Deposition duration (acetone)**				
20 min	120	Good	8.05	123.68
40 min	125	Good	8.10	97.95

**MgCl**_**2**_ **concentration (IPA)**				
0.1 g dm^−3^	117	Good	7.37	89.75
0.2 g dm^−3^	118	Marginal	5.92	58.00

**I**_**2**_ **concentration (acetone)**				
0.2 g dm^−3^	141	Good	8.90	139.00
0.4 g dm^−3^	155	Good	9.50	154.00

**Electrode calendar (acetone)**				
None	155	Good	9.50	154.00
Hot pressed	131	Very good	9.80	165.00

All three solvents have allowed the successful EPD deposition of thick coating layer (109 to 155 μm) and high mass loading (5.92 to 9.80 mg cm^−2^). Whilst NMP and IPA are suitable, thinner deposited coating and low mass loading were recorded. Highest capacitance (165 F g^−1^ @ 0.1 A g^−1^) was found using acetone. It is critical that the solid particles to be deposited has sufficient surface charge (typically zeta potential ± 30 mV) to support their migration to the deposition substrate. In the acetone solvent, it was −35 mV in the absence of iodine as charging agent. When 0.3 g L^−1^ iodine was added, zeta potential changed to +35 mV. The effectiveness of iodine complexing agents (in dry acetone and acetone-water mixtures) in zeta potential manipulation is a consequence of proton formation during acetone iodination, plus water helps adsorption activities; more details about iodine function are available in the published literature.^13^

Stirring of colloidal electrolytes was necessary in order to minimize materials sedimentation and provide hydrodynamic flow in the electrolyte tank. For our series of experiments, 300 rpm stirring was provided by the stirrer bar in the electrolyte tank. For future industrial operations, more controlled fluid flow and scalable stirring of the electrolyte can be deployed, *e.g.* peddling, educator agitation and ultrasonic wave.

The next experiments were focused on understanding the effect of deposition duration (40 min *vs.* 20 min) on coating thickness and mass loading. Favourable thicker coating and higher mass loading were achieved by a longer period of EPD deposition, but this had a detrimental impact on the extracted capacitance (most likely due to electrode limitation challenges, *e.g.* poorer diffusion and tortuous matrix of thick electrode). Growth rate of coating layer was about 6 to 9 μm min^−1^, which is consistent to our previous EPD research on lithium-ion battery electrode manufacture.^1^ For longer deposition, slow-down in growth rate is ideal for depositing uniform thickness all over an irregular 3D complex topography (*e.g.* microporous foam current collectors, fibrous electrodes, 3D printed structures).

Comparative behaviour was found when testing the impact of charging agents (MgCl_2_, I_2_) and their concentration. The limiting effect of concentration on the deposited layer thickness was found, and needs to be adjusted to suit mass loading target. Evidence in the literature suggests that if the deposited layer is porous, which is the case in this study for EPD electrode, the voltage drop across the layer will remain low. The availability of conductance pathways (both ionic and electrical conductivities) through the open porous structure suggests the possibility to electrophoretically deposit an unlimited coating layer thickness from very thin nm to very thick mm scale.^14–16^

It is critical that the colloidal electrolyte is sufficiently stable, offering fast enough deposition whilst ensuring a thick enough coating to provide capacitance for practical energy storage. In this study, all successful deposition was performed by the cathodic EPD approach; so that Al foil would not undergo anodic dissolution. The ability for EPD to use a controllable electric field that directs the travel of charged particles to a deposition surface, which in no doubt, drastically increases its technological applicability to produce controlled electrode structures that is impossible to achieve using the viscous slurry casting that is prone to suspension instability and fast ageing.

### Coating adhesion and microstructures of EPD electrode

**Fig. 3:**
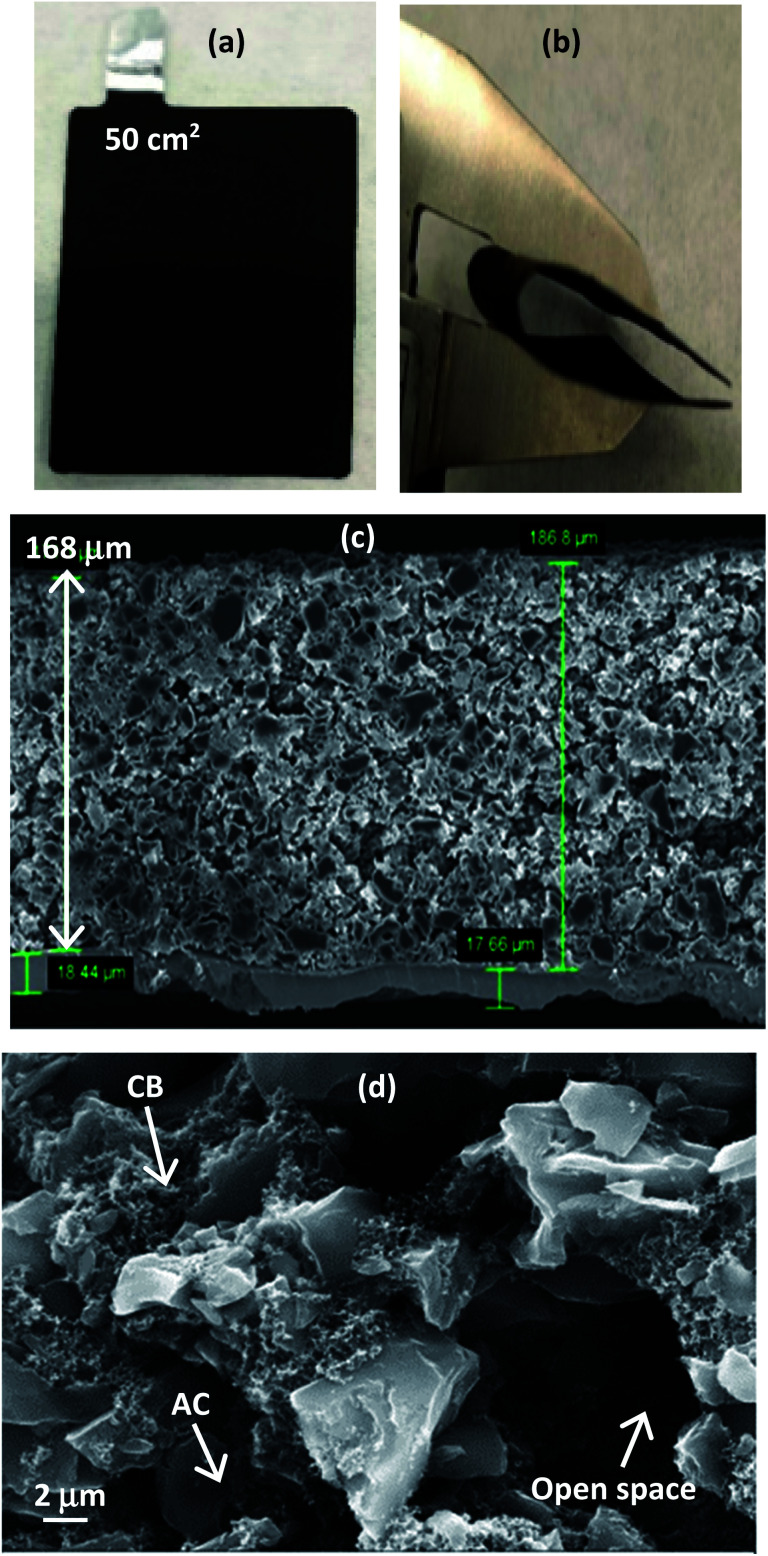
EPD electrode. (a) Actual sample. (b) Bending by calliper. (c) Cross-sectional view. (d) Locations of tiny carbon black (100 to 200 nm) and larger activated carbon (8 to 10 μm), including open space resulted by bubbles evolution (2 to 8 μm).

An example of the large area 50 cm^2^ EPD electrode (used for making pouch cells as described later) is shown in Fig. 3(a). By a simple physical observation, the surface of the Al foil is covered by the deposited materials and no obvious pin-holes can be seen. Following electrode drying and cutting sequences, the electrode continues to show good mechanical integrity with no obvious deposits flaking-off. An extreme bending at 180° using a lab twizzle, see Fig. 3(b), shows no obvious crack lines along the bended section and no delamination.

Qualitative approaches based upon how well the adherence appeared when in contact with supercapacitor electrolyte and physical bending were employed as a quick way to check adhesion properties of EPD electrodes. ‘good’, ‘marginal’ and ‘poor’ terms are used to describe decent adherence (<20% of deposited materials flaking off), not so good adherence (flaking off >30%) and bad adherence (flaking off >50%), respectively. Excellent adherence with <10% flaking off are labelled as ‘very good’, which was the case when EPD electrode went through the hot-pressing step. All these observations clearly suggest the suitability of EPD approach to produce mechanically robust electrodes meeting the targets of industrial manufacturability.

Fig. 3(c) shows cross-sectional image of the EPD electrode. The coating was very thick (168 μm) and had a high mass loading (10 mg cm^−2^); both values meet the commercial suitability as practical electrodes for energy storage. Activated carbon and carbon black can be clearly distinguished by their particle size in the microscopy images. The smaller particle size of carbon black enabled it to infiltrate into the available spacing between the large particles of activated carbon. This strategic placement of carbon black thereby gives the necessary electrical pathways through the entirety of the thick electrode. When 10% carbon black was used, the deposited electrode appears more uniform and displays reasonable porosity. Higher content of carbon black seems to produce denser electrodes, but porosity reduces. High density active materials (90% activated carbon) allows the manufacture of both thin layer for high rate and thick layer for high capacitance, without sacrificing performance by an undesirable quantity of inactive materials.

A closer view of the electrode, see Fig. 3(d), shows an open pore network with tortuosity which extends from top to bottom of the thick electrode, offering beneficial spacing for ions to move readily. During experiments, gas bubbles evolution on the working electrode were observed which likely would have assisted the formation of porosity in the EPD electrodes; this is ideal for porous electrode energy storage applications. The specific surface area of activated carbon was found to be 2000 m^2^ g^−1^ with 0.47 cm^3^ g^−1^ pore volume and 1.1 nm pore diameter. XPS confirmed 90% carbon in the deposit, with hardly any influence from PVDF (no fluorine detected) and minor presence of iodine. In all cases, the porous electrode microstructure and its coating thickness must be optimized to give useful combination of electronic, ionic and interfacial charge transports that maximize the rate at which active materials within the whole electrode can be utilized effectively.

### Electrochemical cycling performance

**Fig. 4:**
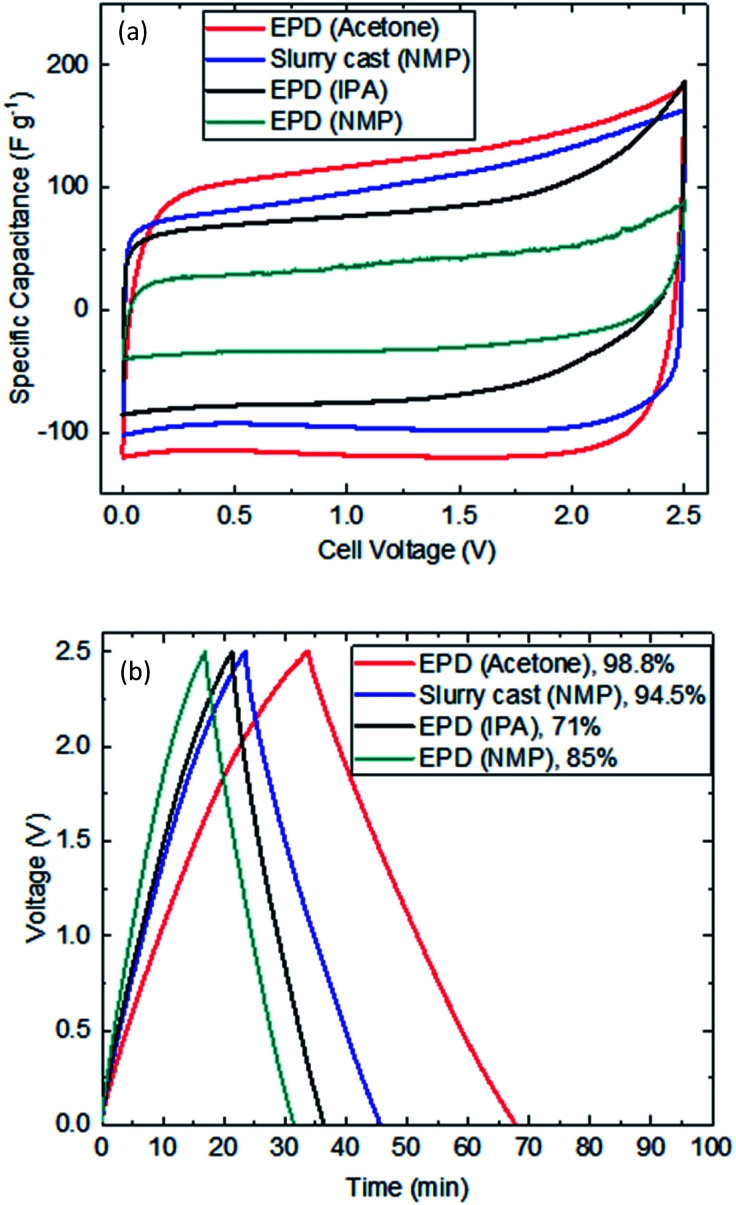
Electrochemical cycling performance of EPD *vs.* slurry cast electrodes. (a) Cyclic voltammetry at 20 mV s^−1^. (b) Galvanostatic testing at 0.1 A g^−1^ between 0 V and 2.5 V. % refers to coulombic efficiency.

Fig. 4(a) shows typical voltammetry response of EPD electrodes. Despite thick layer and high mass loading, clear rectangular shapes representing non-faradaic activity were recorded. Acetone was the best performing electrolyte for EPD electrode manufacture; delivering the highest capacitance (due to the availability of meso to macro porosities that have facilitated better electrolyte penetration, diffusion and migration). EPD electrodes produced by IPA and NMP colloidal electrolytes gave reduced capacitance; the reason is unclear but likely need specific formulations to deliver better performance. Fig. 4(b) shows the galvanostatic response of symmetric coin cells (tenth cycle); clearly showing EPD electrode (acetone) has outperformed slurry cast electrode (NMP) by delivering more capacitance and achieving 98.8% coulombic efficiency. Table 2 summarizes the recorded performance values of EPD (acetone) *vs.* slurry cast (NMP) electrodes.

**Table 2:** Performance advantage of EPD (acetone) *vs.* slurry cast (NMP) electrode. Charge and discharge at 0.1 A g^−1^

Parameters	EPD (acetone)	Slurry cast (NMP)
Discharge time (min)	34	22
Capacitance extracted (F g^−1^)	154	107
Coulombic efficiency (%)	98.8	94.5
Equivalent series resistance (Ω)	0.82	1.11

**Fig. 5:**
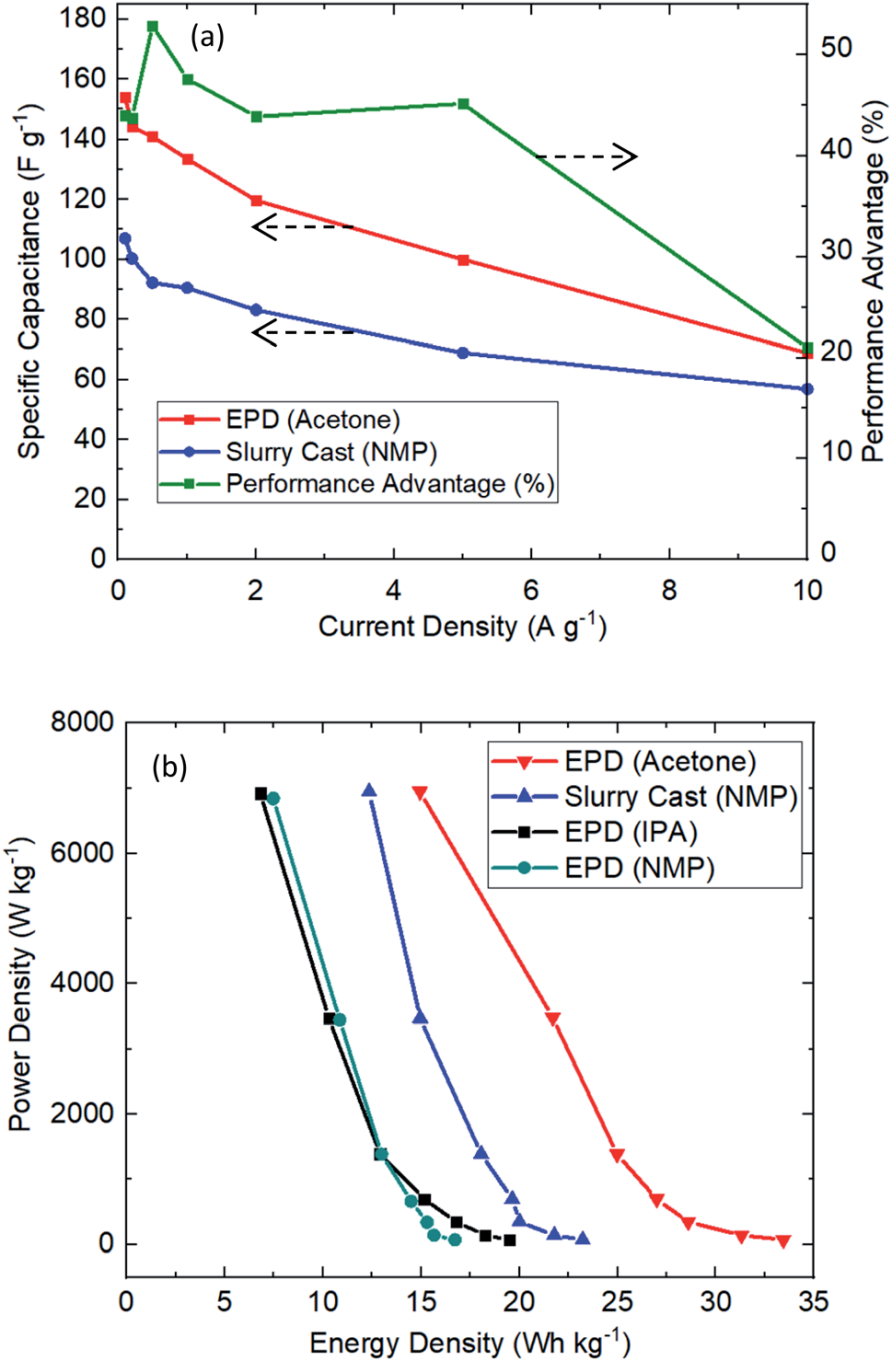
Electrochemical cycling performance of EPD *vs.* slurry cast electrodes. (a) Specific capacitance across various current density. (b) Ragone plot showing power and energy densities.

The extractable capacitance of EPD electrodes were investigated under various current densities, see Fig. 5(a). Clearly EPD electrodes (acetone) have consistently delivered better performance than slurry cast electrodes, including 43% more capacitance extraction, 54% longer use time and 26% lower equivalent series resistance; this is consistently achieved over many current densities (1 to 10 A g^−1^). Fig. 5(b) shows the Ragone plot. EPD electrodes demonstrated useful characteristics for practical applications: high power (7 kW kg^−1^), C-rate (110C) and energy density (33.5 W h kg^−1^). It is worth noting that the extracted capacitance (165 F g^−1^) from EPD electrode (acetone) is very high for activated carbon supercapacitor.

### Electrochemical impedance spectroscopy analysis

**Fig. 6:**
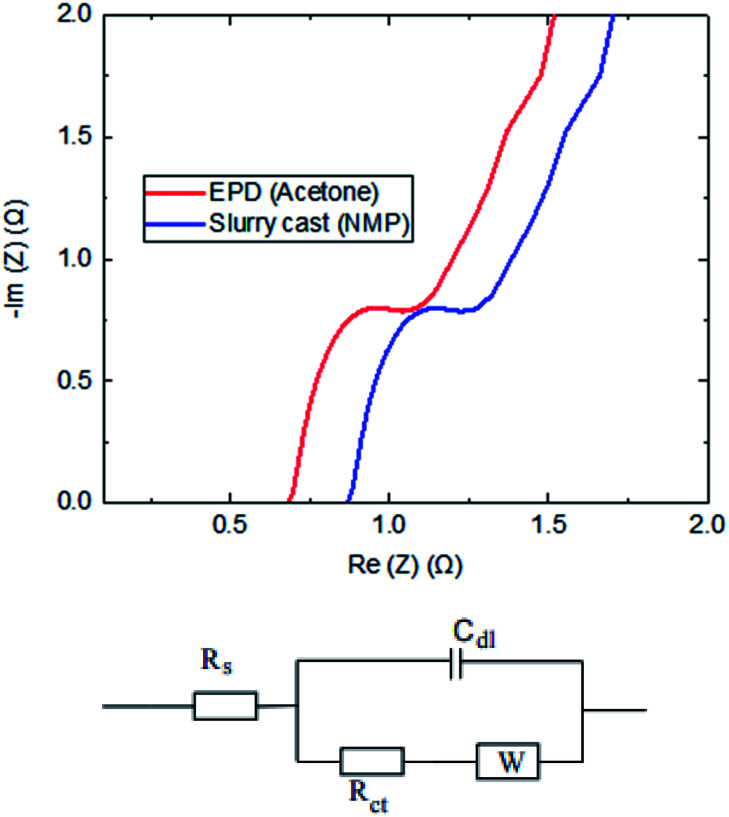
EIS plots comparing electrodes produced by EPD (acetone) and slurry casting. The circuit shows a simple Randles EC model.

The frequency domain response of electrodes was carried out by the Electrochemical Impedance Spectroscopy (EIS) technique. The impedance data were collected at open-circuit potential by applying an alternating potential at a small amplitude (10 mV) over a range of frequencies (0.01 Hz to 10 kHz). A typical example of Nyquist plot is shown in Fig. 6. The semicircles, slopes and *x*-axis intercepts were used to provide interpretation on the recorded resistances and capacitance; consistent to the approaches in the literature.^17,18^ The location of frequencies relates to specific performance:

#### • High to low frequencies (10^4^ to 1 Hz)

Charge transfer resistance associated with electrode porous structure.

#### • Very low frequencies (<1 Hz)

Pure capacitive behaviour. An inclined angle at 45° and 90° would correspond to Warburg and ideal capacitive diffusion.

Since an ideal capacitor does not exist in real supercapacitor devices, a simple Randles equivalent circuit model was used to interpret the combination of kinetic and diffusion processes in EIS data. It includes a solution resistance (*R*_s_), a charge transfer resistance (*R*_ct_), double layer capacitance (*C*_dl_) and Warburg diffusion (*W*). The value of capacitance was not analysed using EIS data, as some believe that their interpretation can be erroneous by approximately 20%.^19,20^ Values of the resistances, which are represented by points where the spectrum crosses the *x*-axis, are seen to vary significantly. Clearly, the EPD electrodes are much superior *vs.* slurry cast electrodes, which was validated by the winning characteristics of lower resistance.

#### • Solution resistance (*R*_s_)

This is taken from the first point with *x*-intercept. 0.68 Ω (EPD) < 0.87 Ω (slurry cast).

#### • Charge transfer resistance (*R*_ct_)

This is the width of semi-circle on *x*-intercept. 1.25 Ω (EPD) < 1.47 Ω (slurry cast).

This is unsurprising considering the controlled porous structure in the EPD electrode, which contains strategic placement of tiny carbon black particles around much larger size of activated carbon particles in a thick coating. This is consistent to some earlier studies which have shown manipulation of the particle size, surface area and porosity as ways to improve supercapacitor performance.^21^

Total resistance of the supercapacitor based on slurry cast electrode (NMP) was 1.74 Ω, whilst much reduced resistance on EPD electrode (acetone) around 1.31 Ω was recorded; this is 25% reduction in total resistance EPD *vs.* slurry cast. When the frequency decreases, the real capacitance sharply increases, then tends to be less frequency dependent. The reciprocal of the frequency at which the imaginary capacitance shows a slope, known as the response time (time constant), is another important performance parameter. The time constant for slurry cast electrode (NMP) supercapacitor discharge is 15.4 seconds, whilst that for the EPD electrode (acetone) supercapacitor discharge is 9.5 seconds; showing EPD electrode provides more discharge power than slurry cast counterparts.

### Electrode calendaring and cycling robustness

**Fig. 7:**
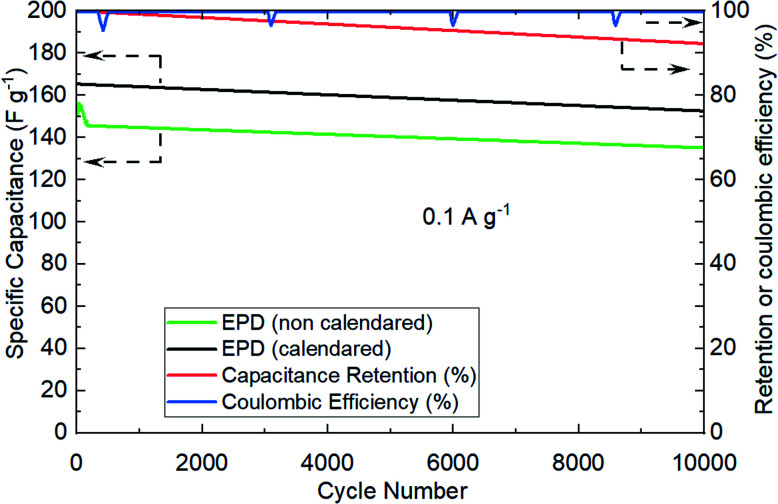
Cycling robustness of EPD electrodes showing calendared *vs.* non-calendared performance. Specific capacitance (left *y*-axis). Retention and coulombic efficiencies for calendared EPD electrodes (right *y*-axis). 0.1 A g^−1^ was used. Coin cell format (CR2032).

The next experiments were focused on understanding the effect of electrode calendaring on cell performance, and measuring their cycling robustness in terms of coulombic and retention efficiencies. Fig. 7 shows an example of the recorded performance, specifically comparing calendared *vs.* as-prepared electrodes. The coin cells were cycled repetitively at 0.1 A g^−1^ over 10 000 cycles. Clearly, calendared electrodes allows more capacitance extraction (about 8% better) *vs.* non-calendared electrodes. This observation is consistent to the published evidence in lithium-ion battery,^22^ where electrode calendaring step would have contributed to improve the physical and electrical contacts between particles and current collector surfaces.

Cycling robustness of EPD electrodes were demonstrated by the minimal reduction in capacitance over successive cycling, showing high retention efficiency (95%) and coulombic efficiency (100%).

### Pouch cell cycling performance

An early evidence of the promising industrial application of EPD electrodes was the final investigation. The practical EPD electrodes were manufactured onto a larger scale (50 cm^2^) surface area; the equipment setup and operation were similar to prior art, but deployed a slightly bigger electrolyte volume (600 cm^3^) which was sufficient to minimise significant impacts from composition swings during experiments.

It is recognised that the electrolyte composition (*e.g.* mass ratio of activated carbon to carbon black, binder content, pH, zeta potential) may change during EPD operation and dependent on the finished product (*e.g.* deposited surface area, mass loading and coating thickness). An imbalanced electrolyte composition would lead to an undesirable finished product. Accurate monitoring and maintenance of colloidal electrolyte composition are therefore crucial in view of producing good quality EPD electrode. Quality control tools such as Hull cell^23^ and analytics such as electrolyte turnover rate and throwing power,^24^ which are practiced by the electroplating industry, are translatable knowledge for successful EPD electrode manufacture and their operation with industrial relevance. We are now researching these themes for the next stage development in the industrialisation of EPD energy storage electrodes.

**Fig. 8:**
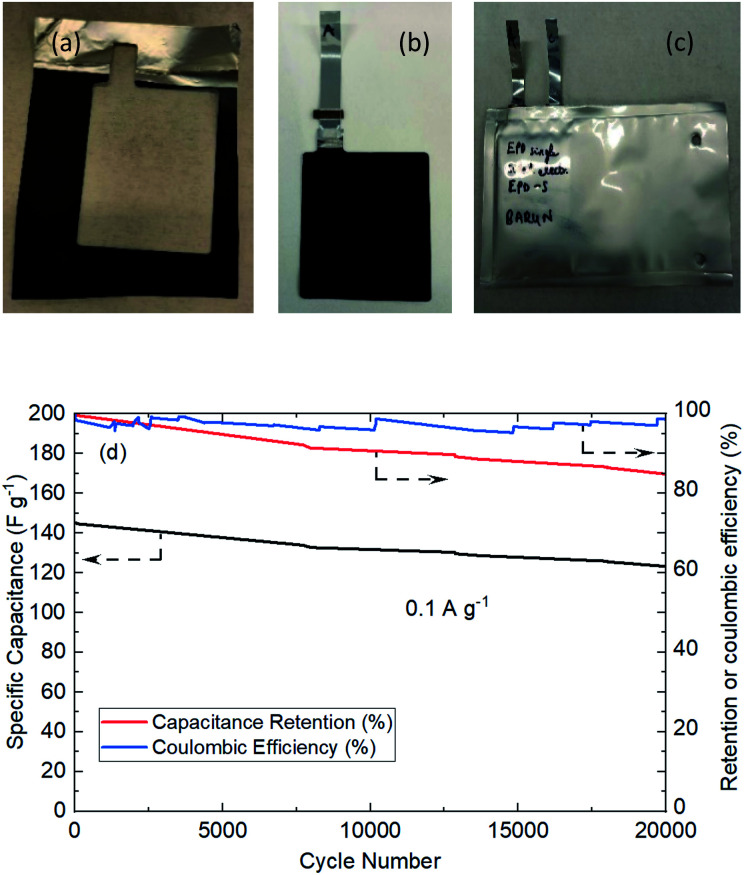
Pouch cell manufacture and testing. (a) A8 electrode area was stamped out. (b) Current collector tag welded to the Al foil. (c) Completed A7 pouch cell build. (d) Pouch cell cycling performance.

Fig. 8 shows the actual photos of large area EPD electrode in (a), which was stamped out to A8 dimension and welded to current collector tag in (b), and assembled into pouch cell in (c). No obvious electrode delamination or flaking-off deposits were seen at the edges of stamping. The EPD electrode was successfully processed through the industrial machinery tools for pouch cell assembly.

The cell was cycled at 0.1 A g^−1^ for both charge and discharge cycles in Fig. 8(d). Clearly, the specific capacitance of the pouch cell was comparatively high (drops from 145 to 123 F g^−1^ in 20 000 cycles) *vs.* those in published reports. The pouch cell was only showing about 12% less capacitance than coin cell; the retention efficiency was reducing (but still above 85% meeting industry target) and coulombic efficiency was maintaining close to 100%. Fundamental reasoning behind the observation was unclear at this stage, but it is recognised that pouch cell cycling performance could be further improved by deploying the scientific principles and practices of electrochemical engineering reactors for EPD electrode manufacture and operation, *e.g.* improving mass transport, maintaining uniform distribution, controlling electrolyte composition; plus fundamental studies to understand electrode properties, *e.g.* tortuosity of porous structure, electrochemistry of faradaic phenomena in activated carbon.

### Comparing solvent choice of EPD (acetone) *vs.* slurry cast (NMP)

A simple solvent comparison was performed between EPD (acetone) and slurry casting (NMP). According to the prices from Sigma-Aldrich, acetone (£30 g L^−1^) costs much less than NMP (£93 g L^−1^); this would be much lower in bulk commercial quantity. The solid content can vary from 1 to 100 g L^−1^ in acetone EPD operation; whilst much viscous 50 to 70 wt% in NMP slurry casting. A drying line is needed to evaporate off NMP solvent from within the electrode layer, hence an expensive recovery line is needed to collect the evaporated solvent. Acetone lift-off only needs low to moderate 40 to 50 °C heating; whilst NMP is a very slow evaporating solvent (it has a boiling point around 202 °C). For very thick electrode (100 to 200 μm), it would obviously take much longer to complete the electrode drying and solvent recovery steps.^25,26^

Nonetheless, NMP has a higher flash point (91 °C) *vs.* acetone (−20 °C) thus offering safety advantage, plus lower vapour pressure means lower volatile organic compound emissions. But, NMP is identified as a reproductive hazard and associated with legislations for usage restriction in European Union and other continents. The hazardous use of acetone is related to its low flash point, where its vapour can flow along surfaces to distant ignition sources and flash back. But, it has a high auto initiation temperature (465 °C). Acetone use must be in a well ventilated environment. It is noted that industrial acetone contains water, which helps to inhibit ignition, and is widely used in the textile industry for degreasing wool and degumming silk. It is a common solvent in plastics and industrial processing, plus household products such as personal care cosmetics (*e.g.* nail polish remover). Because acetone is an organic compound, it is non-toxic to animals and the environment.

## Conclusions

The energy storage electrode performance is greatly dependent on the active material mass loading and layer thickness. It is misleading to take advantage of the high capacity (in the case for battery) or high capacitance (in the case for supercapacitor) by low mass loading and thin layer to compare with commercial devices, which needs high mass loading (5 to 20 mg cm^−2^) and thick layer (50 to 200 μm). This is especially exacerbated in electrophoretic deposition (EPD) research and innovation, for energy storage electrode manufacture, where the literature only reports low mass loading of active materials and thin layer coating. The research in this study, is therefore, showing the evidence needed to close this knowledge gap for industrial relevance, specifically by manufacturing and testing practical EPD electrodes that show characteristics of high mass loading and thick layer; thus making them commercially suitable for high power and energy density supercapacitor devices.

It was found that activated carbon supercapacitor electrodes prepared by EPD can reach useful layer thickness (168 μm) and high mass loading (10 mg cm^−2^), giving high capacitance (165 F g^−1^). The manufacture of practical EPD electrodes was successful through controlled manipulation of colloidal electrolyte recipes, deposition parameters and post process calendaring step.

Very high power capability (7 kW kg^−1^), C-rate extraction (110C) and energy density (33.5 W h kg^−1^) were recorded on EPD electrodes for supercapacitors. Compared with slurry cast, EPD electrodes have demonstrated many performance advantages including 43% more capacitance, 54% longer use time and 26% lower equivalent series resistance; this is consistently achieved over a wide range of current densities (1 to 10 A g^−1^). Tiny carbon black particles (100 to 200 nm) were distributed around the bigger activated carbon particles (8 to 10 μm) and an open porous network (2 to 10 μm); all have contributed to give useful combination of electronic, ionic and interfacial charge transports that maximize the rate at which materials within the practical electrode can be utilized effectively.

Cycling robustness of EPD electrodes (over 10 000 cycles) were shown by high retention efficiency (95%) and coulombic efficiency (100%). The translation from coin cells to pouch cells (A7 size) was successful. Pouch cells had only 12% less capacitance than coin cell; the retention efficiency was reducing (still over 85%) but coulombic efficiency was maintained constantly close to 100%. The colloidal electrolytes were formulated by acetone–water–iodine mixtures with suitable PVDF binder system. The use of acetone, low drying temperature, effectiveness of iodine complexes in acetone and water assisted adsorption are characteristics of a suitable solvent for EPD operation with industrial compatibility, which can be suitably applied as a platform electrode manufacturing process technology for any materials with practical applications in lithium-ion batteries, supercapacitors and solid-state devices.

## Author contributions

Low secures the project funding and provides research leadership. Chakrabarti performs the experiments and produces testing data. They both contributed to the results discussion and their analysis, plus write-up manuscript for submission to the journal.

## Conflicts of interest

There are no conflicts to declare.

## Data availability

Data underlying this paper may be accessed from the authors upon request.

## Supplementary Material

RA-011-D0RA09197A-s001
