# Supplementary material for: Practical aspects of electrophoretic deposition to produce commercially viable supercapacitor energy storage electrodes
Source: RSC Adv. 2021 Jun 9;11(34):20641–50. doi: 10.1039/d0ra09197a (PMC9033947; doi:10.1039/d0ra09197a)
Supplement: RA-011-D0RA09197A-s001 [file RA-011-D0RA09197A-s001.pdf]

Electronic Supplementary Material (ESI) for RSC Advances.
This journal is © The Royal Society of Chemistry 2021

Supporting Information

Practical Aspects of Electrophoretic Deposition to Produce Commercially Viable Supercapacitor Energy Storage Electrodes

Barun Kumar Chakrabarti, Chee Tong John Low
WMG, Warwick Electrochemical Engineering Group, Energy Innovation Centre, University of Warwick, Coventry, CV4 7AL, United Kingdom.

Barun.Chakrabarti@warwick.ac.uk (Dr. Barun Kumar Chakrabarti)
C.T.J.Low@warwick.ac.uk (Dr. Chee Tong John Low)

1. Activated carbon material properties

Material properties	Values
Activated carbon brand name	YEC-8B
Manufacturer	Fuzhou Yihuan Carbon (China)
Surface Area (m^2 g^{-1})	2000 to 2500
Ash (%)	< 0.5
Moisture (%)	<5
Bulk Density (g mL^{-1})	0.4
Capacitance with PC (F g^{-1})	140
Capacitance with KOH (F g^{-1})	260

Table S1. Specification of activated carbon used in this research study.

2. EPD operation and setup

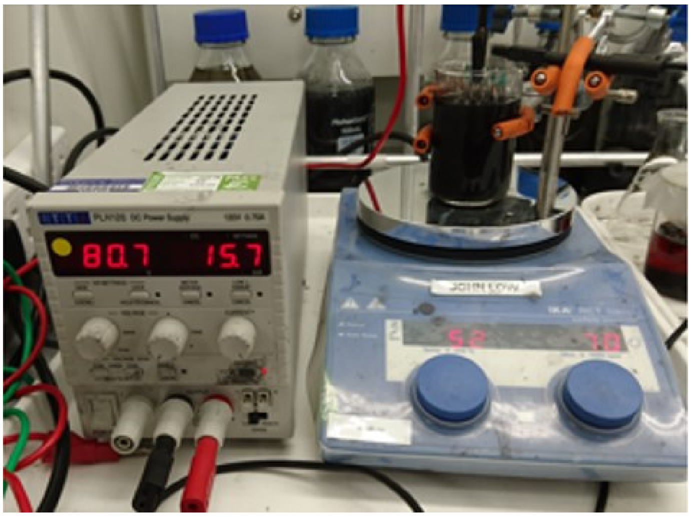

10 cm

Figure S1. A simple beaker version of electrochemical cell setup. Power supply rating (max 250 V, 750 mA) and beaker volume (max 100 mL). Colloidal electrolyte was kept under 300 rpm and 40 °C.

3. EPD electrodes in coin cell

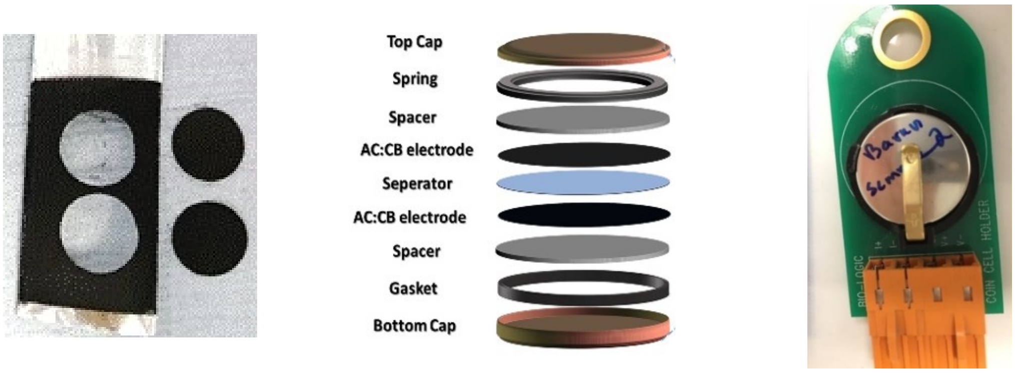

Figure S2. EPD electrodes in coin cell (CR2032 format). Symmetric coin cell setup was assembled.

4. BET and pore size of activated carbon particles

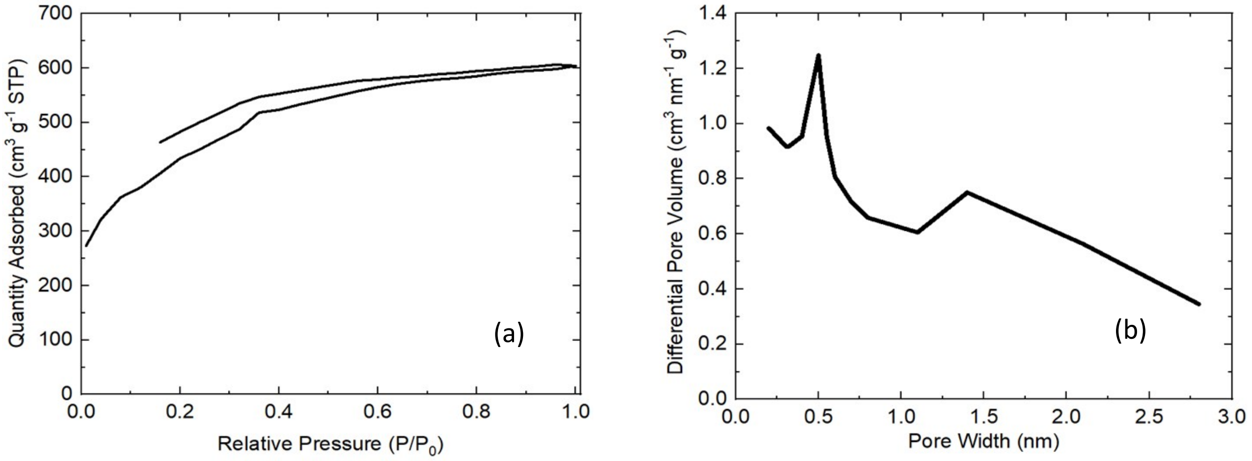

Figure S3. Material properties of activated carbon particles. (a) BET adsorption/desorption isotherms. (b) Pore size distributions (NLDFT). Specific surface area of activated carbon was determined to be 2000 $m^2 g^{-1}$ along with 0.47 $cm^3 g^{-1}$ pore volume and 1.1 nm pore diameter.

5. X-ray photoelectron spectroscopy analysis of EPD electrode

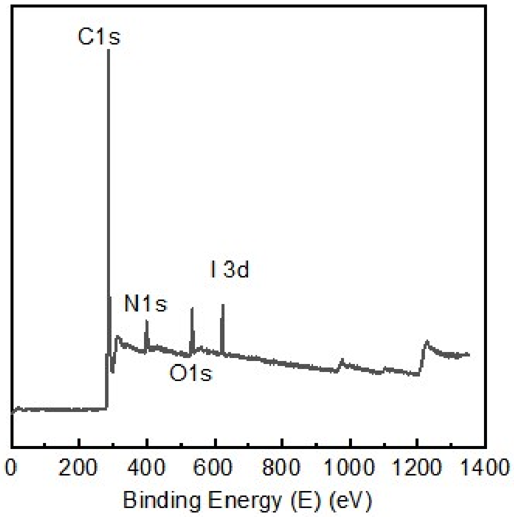

Figure S4. XPS analysis of EPD electrode; detection in the top 3 to 30 atomic layers (10 to 100 Å). The majority 90% is carbon element; no fluorine detected and minor presence of iodine.

6. Elemental composition mapping analysis of EPD electrode

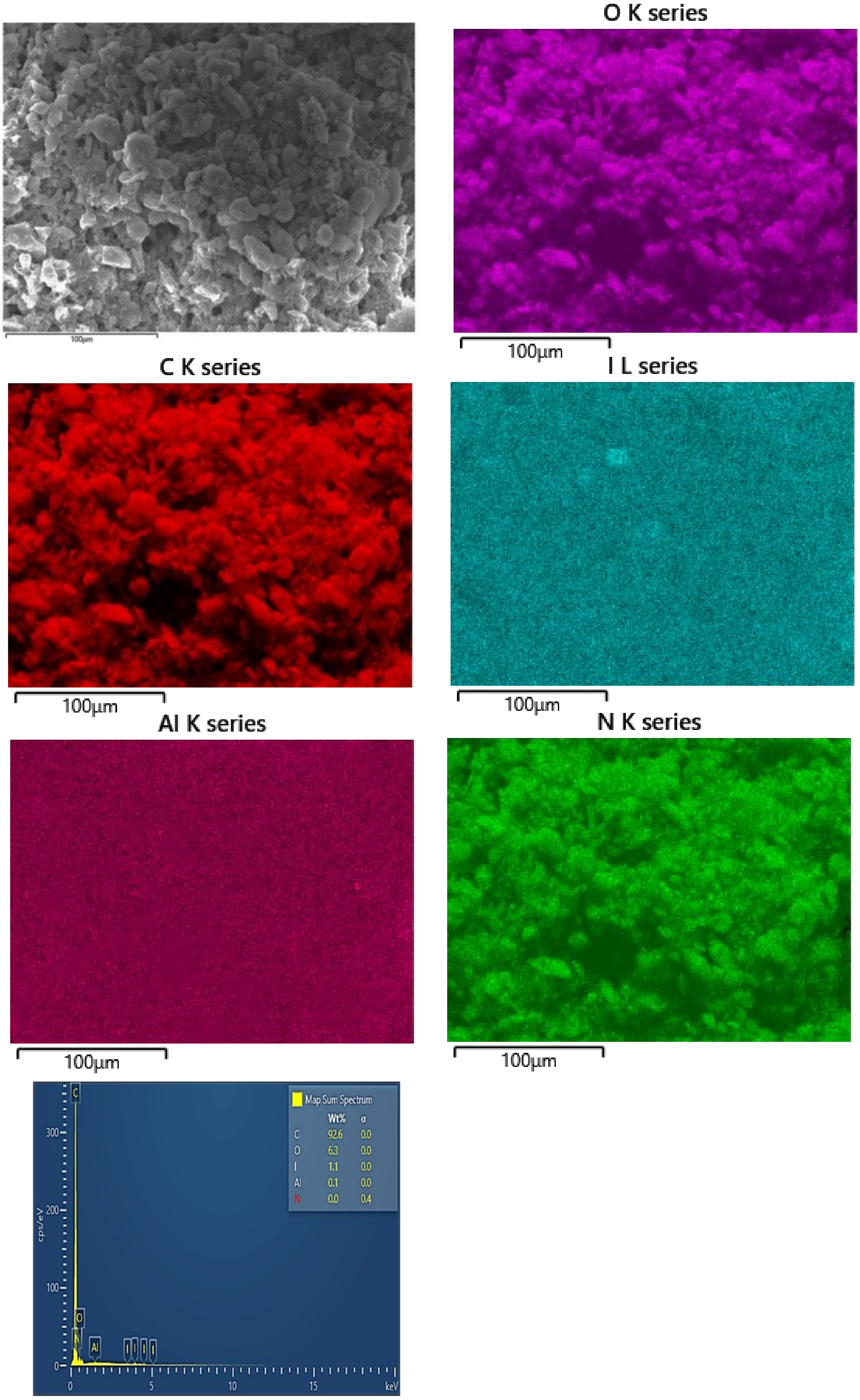

Figure S5. EDX mapping of EPD electrodes showing fluorine is undetectable (PVDF indication) and minor presence of iodine resulted from electrophoretic deposition. The EPD electrode was used as it is, i.e. no washing off with any liquid prior assembly into coin cell. The presence of iodine could be resulted from the charging agent inclusion during deposition, as well as left-over solution from non-washed electrode.

7. Cyclic voltammetry response of electrodes (EPD vs slurry cast)

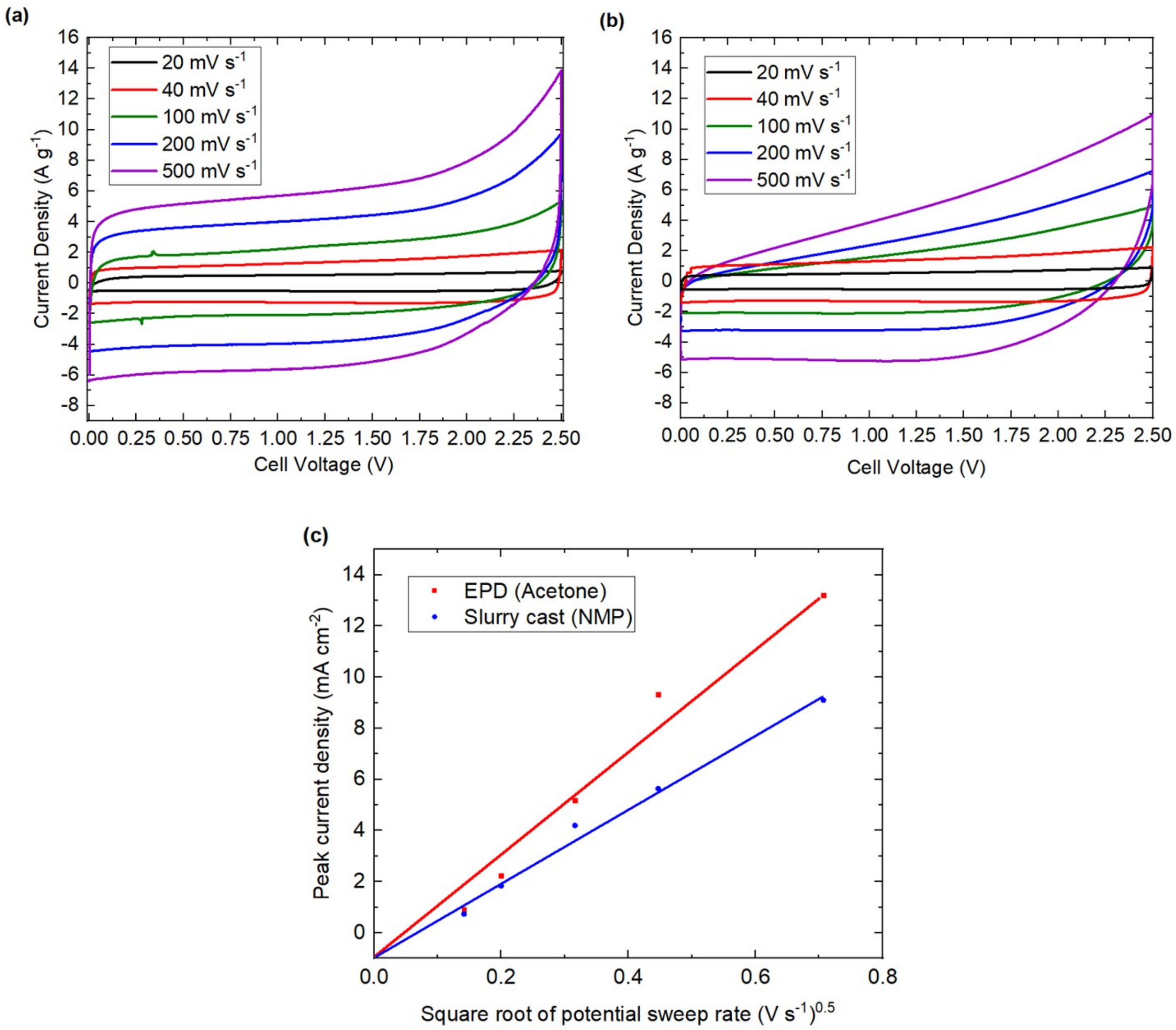

Figure S6. Applied voltage vs recorded current response of deposited electrodes. (a) EPD electrode using acetone recipe. (b) Slurry cast electrode using NMP recipe. (c) Randles-Servick plot at 1.5 V to determine diffusion coefficient of ionic species.

Diffusion coefficient for EPD (Acetone) from Figure S6(c) = 9.4×10^{-6} cm^2 s^{-1}
Diffusion coefficient for slurry cast (NMP) from Figure S6(c) = 2.3×10^{-6} cm^2 s^{-1}
Almost 5 times higher diffusion rate for EPD (Acetone) vs Slurry cast (NMP).

Current density (A/g)	1Specific capacitance (F g^{-1})		Performance advantage (%)
	EPD (Acetone)	Slurry cast (NMP)	
0.1	154.08	107.00	44
0.2	144.25	100.35	44
0.5	140.97	92.20	53
1	133.59	90.50	48
2	119.66	83.17	44
5	99.99	68.89	45
10	68.84	56.91	21

Table S2. Recorded performance values for Figure 5.

8. Galvanostatic cycling of electrodes and Ragone plot (EPD vs slurry cast)

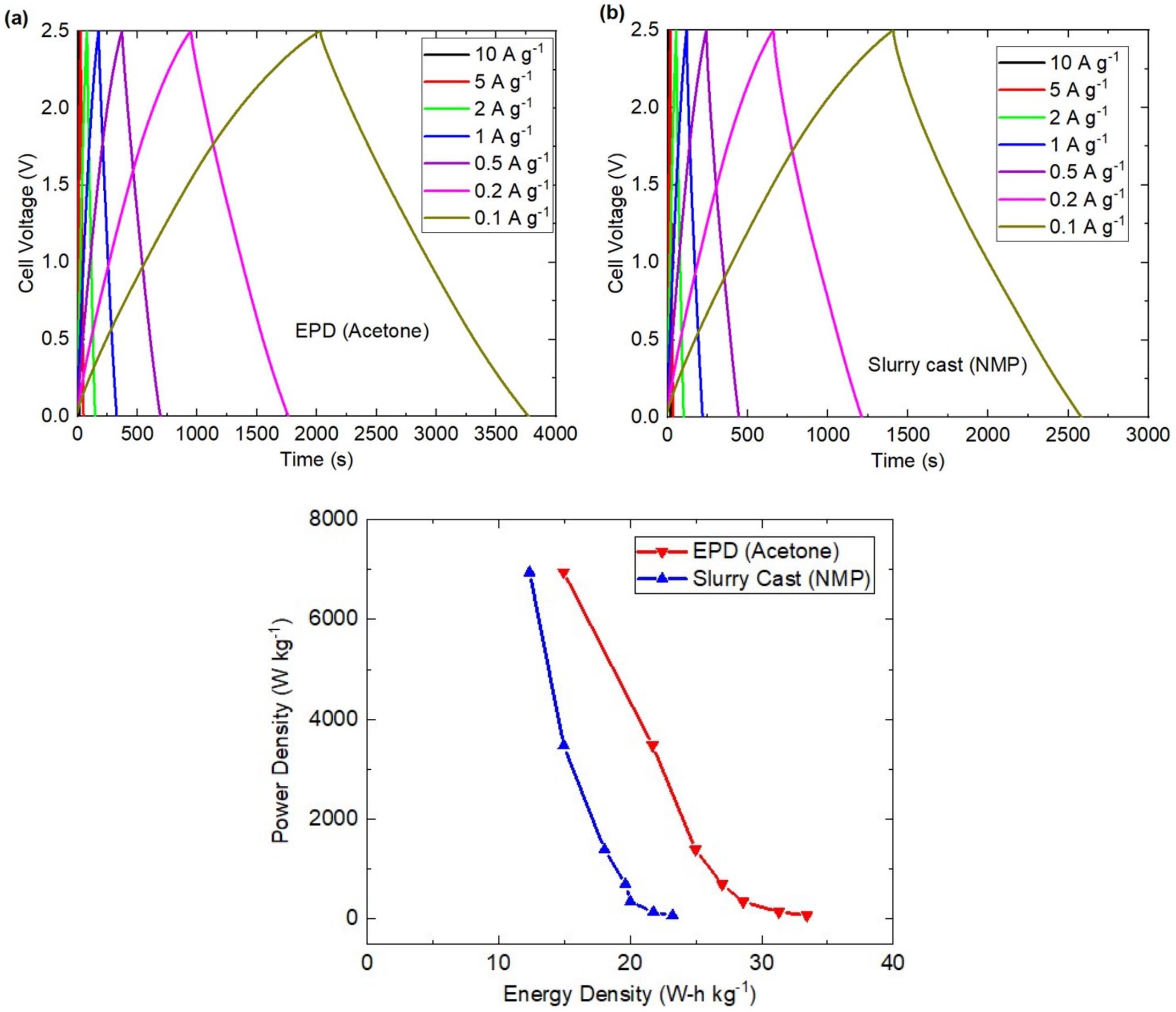

Figure S7. Constant current cycling and comparing data. (a) EPD electrode using acetone recipe. (b) Slurry cast electrode using NMP recipe. (c) Ragone plot showing data from both electrodes.

EPD (Acetone)		Slurry cast (NMP)		Performance advantage based on energy density gained in EPD vs slurry cast electrode (%)
Energy density ($W\ kg^{-1}$)	Power density ($Wh\ kg^{-1}$)	Energy density ($W\ kg^{-1}$)	Power density ($Wh\ kg^{-1}$)	
33.44	69.99	23.22	69.44	44
31.30	138.89	21.78	138.89	44
30.59	347.22	20.01	347.22	53
28.99	694.44	19.64	694.46	48
25.97	1388.84	18.049	1388.89	44
21.70	3479.54	14.95	3472.11	45
14.94	6948.91	12.35	6944.72	21

Table S3. Recorded performance values for Figure S7.
